# The Differences in Clinical Manifestations and Prognosis of Infective Endocarditis Patients With Positive Serology to Antineutrophilic Cytoplasmic Antibody Compared to Negative Serology

**DOI:** 10.7759/cureus.51211

**Published:** 2023-12-28

**Authors:** David J Ozeri, Shani Peretz, Bianca Brif, Itai Gueta, Amit Oppenheim

**Affiliations:** 1 Rheumatology, Sheba Medical Center, Ramat Gan, ISR; 2 Internal Medicine A, Sheba Medical Center, Ramat Gan, ISR; 3 Infectious Disease, Sheba Medical Center, Ramat Gan, ISR

**Keywords:** valvular endocarditis, high mortality, glomerular nephritis, infective endocarditis, antineutrophil cytoplasmic antibody (anca)

## Abstract

Previous studies have established a relationship between bacterial proteins and autoimmune diseases through several mechanisms. Infective endocarditis is known for its immunological phenomena, and the presence of antineutrophil cytoplasmic antibodies (ANCA) antibodies has been previously demonstrated in several infectious diseases. This retrospective, comparative, and descriptive study examined the relationship between infective endocarditis and the presence of ANCA antibodies.

Ninety infective endocarditis cases were included in the study and tested for ANCA antibodies. The prevalence of ANCA positivity was determined, along with the differences in characteristics and prognosis between infective endocarditis patients with positive and negative serology for ANCA antibodies. The results showed that the characteristics of endocarditis patients who underwent ANCA serology testing were similar to those who did not, except for a higher prevalence of central line and chronic kidney disease in patients with ANCA serology (6.7% compared to 1.1% and 25.6% compared to 12.9%, respectively).

Of the 90 endocarditis patients tested for ANCA serology, 18% were ANCA-positive, consistent with other prospective studies. There were no statistically significant differences in the primary outcome, six-month and one-year mortality, between patients with positive and negative ANCA serology. Similarly, in the secondary outcomes of acute kidney injury, heart surgery, and days of hospitalization, there were no statistically significant differences between patients with positive and negative ANCA serology.

However, there were statistically significant differences in certain characteristics between the two groups. Patients with positive ANCA serology were found to have a higher prevalence of Enterococcus involvement (29.4% compared to 9.6% with P-value 0.046) and Q fever (23.5% compared to 4.1% P-value 0.02%). In contrast, patients with negative ANCA serology had a higher prevalence of fever (73% compared to 41% P-value 0.033).

## Introduction

Infectious endocarditis is a disease that affects the heart's endothelial tissue, usually causing infection in the heart valve or intracardial devices [1]. Its prevalence is more common now than in the past, with its incidence in the United States increasing from 9.3 per 100,000 population in 1998 to 15 per 100,000 in 2011 [2]. The in-hospital mortality for infective endocarditis is approximately 20%, and the six-month mortality is about 30% [3].

Immunological phenomena, including Osler's node, Roth's spots, and elevated levels of rheumatoid factor, are well known to be associated with the disease [4].

Antineutrophil cytoplasmic antibodies (ANCA) are self-antibodies, mainly of the IgG type, that bind to neutrophil granules and monocyte lysosomes. ANCA antibodies are attached to multiple antigens, with two relevant antigens being proteinase 3, which characterizes the cytoplasmic painting pattern (C-ANCA), and myeloperoxidase, which characterizes the perinuclear painting pattern (P-ANCA) [5]. ANCA antibodies activate neutrophils by enhancing their movement ability, their attachment to the endothelium, and by releasing proteolytic enzymes and proinflammatory cytokines. ANCA and, more specifically, the enzymes myeloperoxidase (MPO) and proteinase 3 (PR3) are found in autoimmune vasculitis, which is known as ANCA-associated vasculitis and includes granulomatosis with polyangiitis, microscopic polyangiitis, and eosinophilic granulomatosis [6]. Nonetheless, these antibodies are also found in numerous infectious diseases, including viral, bacterial, fungal, and parasitic infections [7].

Several hypotheses have been proposed regarding the relationship between bacterial proteins and autoimmune diseases. These include molecular mimicry between human lysosome-associated membrane protein-2 and the bacterial protein FimH [8]. The indirect complementary antisense bacterial RNA proteins create an interaction with C-ANCA antibodies, where the sense and antisense sequences are attracted to each other and create immune complexes that cause the disease [9]. In toll-like receptor (TLR) activation, many bacterial organisms carry hypomethylated CpG motifs in their DNA that activate TLR 9, and it is known that in vitro exposure to hypomethylated CpG activates ANCA creation, especially C-ANCA [10]. Epigenetic changes influence ANCA serology [11]. Neutrophil extracellular traps (NETs) capture chromatin, citrullinated histone H3, and antibacterial proteins such as ANCA antibodies that entrap and kill bacteria. The binding of neutrophils with ANCA antibodies can create these NETs [12].

There are initial reports in the literature about the correlation between ANCA antibodies and infectious endocarditis. These reports present a prevalence of 24% of positive ANCA antibodies in infectious endocarditis patients. Initial reports on small groups including 50 patients exhibited different clinical characteristics in patients with positive and negative ANCA antibodies. A research study from 2014 showed that infectious endocarditis patients with positive ANCA antibodies were younger, fulfilled Duke's criteria more frequently, and had a higher prevalence of vegetation in echocardiography [13-16]. In contrast, a study from France in 2015 found different characteristics such as a higher prevalence of weight loss, kidney involvement, a longer time between symptoms and diagnosis, a higher prevalence of two valves, and lower rates of CRP [16].

## Materials and methods

In this retrospective case-control study, we aimed to investigate and describe cases of infectious endocarditis at Sheba Medical Center from January 2010 to July 2020. The study population included patients aged 18-80 who received a diagnosis of infectious endocarditis. Cases were identified using MDClone software. MDCLone is a healthcare data platform that allows precise preliminary case selection using predetermined criteria.

**Figure 1:**
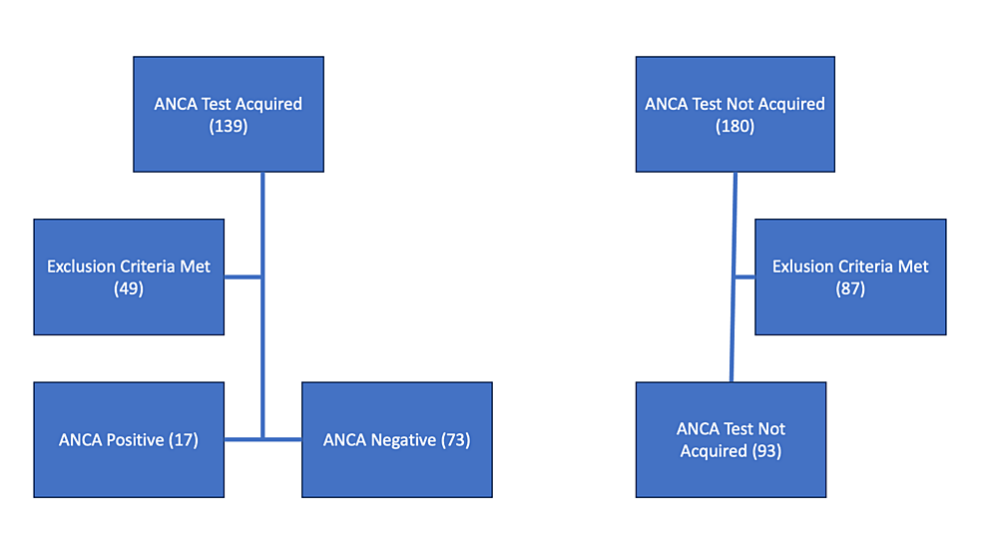
Flowchart depicts subjects included in the analysis ANCA: antineutrophil cytoplasmic antibodies

In our study, cases were identified cases of infective endocarditis using the International Classification of Diseases, Ninth Revision, Clinical Modification (ICD-9-CM). To ensure a comprehensive understanding of each case, we employed a two-step case selection process. Subsequently, each selected case underwent a meticulous manual examination to guarantee the accuracy and completeness of the extracted data. Cases where infective endocarditis was not confirmed were excluded from the analysis (Figure 1).

Once cases were identified and extracted, further patient data was also extracted and analyzed. Data collection involved the extraction of detailed information, including demographic data, medical history, ANCA serology, and other clinical parameters, from each case. Statistical analyses were performed using IBM SPSS software. Categorical variables were subjected to the Fisher exact test, while continuous variables underwent analysis using the Mann-Whitney U-test. A significance level (alpha) of 0.05 was chosen as the threshold for statistical significance, striking a balance between minimizing type I errors and ensuring the detection of clinically meaningful associations. Multivariable analysis was conducted through logistic regression to explore the simultaneous impact of multiple variables on the outcome of interest. The selection of variables for the regression model was based on their clinical relevance and statistical significance in single-variable analyses.

This study was approved by the Ethics Committee at Sheba Medical Center.

## Results

Out of 139 patients with infectious endocarditis who had ANCA antibodies taken, only 90 were included in this study, of whom 73 had negative ANCA serology (81.1%) and 17 had positive ANCA serology (18.8%). A control group of 180 patients who did not have ANCA serology taken was also examined, of which 93 patients were included in the study. The flowchart in Figure 1 shows the groups.

There were no significant differences in age and sex between patients who were tested for ANCA antibodies (ANCA+, ANCA-) and those who were not tested (ANCA NT). Patients who were tested for ANCA antibodies were less dependent (16.7% compared to 20.4%), had a lower prevalence of diabetes mellitus (32% compared to 36%), and had a higher prevalence of central line (6.7% compared to 1.1%) and chronic kidney disease (25.6% compared to 12.9%).

Among patients who had ANCA antibodies taken, there was a higher prevalence of men in ANCA-negative patients compared to ANCA-positive patients (72.6% compared to 64.7%). There was a higher prevalence of dependent patients in ANCA-positive patients (23.5% compared to 15.1%) and a higher prevalence of chronic kidney disease in ANCA-positive patients (35.3% compared to 23.3%) (Table 1).

**Table 1:** Clinical characteristics of patients with infective endocarditis

ANCA Acquired (90)						ANCA Not Acquired (93)	
ANCA Negative (n=73)			ANCA Positive (n=17)		Total Sum of ANCA Acquired (90)		
		%	Number / Mean ± Standard Deviation	%		Number / Mean ± Standard Deviation	%
Age	65.1±15.3		65.5 ± 11.2		65.2 ± 14.5	64.9 ± 17.0	
Male	53	72.60	11	64.70	71.10	69	74.20
Dependent Functional State	11	15.10	4	23.50	16.70	19	20.40
Hypertension	37	51.40	8	47.10	50.00	48	51.60
Diabetes	23	31.50	6	35.30	32.20	34	36.60
Central line	5	6.80	1	5.90	6.70	1	1.10
Preceding Surgery (1 Month)	2	2.70	0	0.00	2.20	9	9.90
Chronic Kidney Disease	17	23.30	6	35.30	25.60	12	12.90
Immunosuppression	5	6.80	1	5.90	6.70	5	5.40
Prosthetic valve	29	39.70	6	35.30	38.90	36	39.10
Pacemaker	11	15.10	3	18.80	15.60	28	30.10
Intravenous Drug Use	0	0.00	0	0.00	0.00	2	2.20
Oral Procedure Preceding (1 Week)	4	5.50	3	17.60	7.80	3	3.30
Valvular Predisposition	39	54.20	11	64.70	55.60	50	54.30
Prior Endocarditis	6	8.20	1	5.90	7.80	6	6.60
Known Vasculitis	1	1.40	1	5.90	2.20	0	0.00
Sinusitis	0	0.00	2	11.80	2.20	0	0.00
Asthma	2	2.70	0	0.00	2.20	0	0.00

With regard to bacterial characteristics, patients with positive ANCA antibodies had a higher prevalence of *Enterococcus *involvement (29.4% compared to 9.6%, P-value 0.046) and a higher prevalence of Q fever involvement (23.5% compared to 4.1%, P-value 0.022). Patients with negative ANCA antibodies had a higher prevalence of fever (73% compared to 41%, P-value 0.033). Other clinical characteristics were not statistically significant (Table 2). There were no significant differences in the valves involved between patients with positive and negative ANCA antibodies (Table 3).

**Table 2:** Comparison of endocarditis causing microbial pathogens ANCA: antineutrophil cytoplasmic antibodies; HACEK Group: *Hemophilus* species, *Aggregatibacter* species, *Cardiobacterium hominis*, *Eikenella corrodens*, and *Kingella* species

	ANCA-negative (N=73)		ANCA-positive (N=17)		
Microbiology	Frequency	Percent	Frequency	Percent	p-value (Fisher's exact test)
Staphylococcus	20	27.4	2	11.8	0.224
Streptococcus	30	41.1	3	17.6	0.095
Enterococcus	7	9.6	5	29.4	0.046
HACEK Group	3	4.1	1	5.9	0.574
Q Fever	3	4.1	4	23.5	0.022
Other	5	6.8	0	0.0	0.579
Unknown	5	6.8	2	11.8	0.613

**Table 3:** Comparison of the heart valve involvement in ANCA-negative and ANCA-positive patients ANCA: antineutrophil cytoplasmic antibodies

	ANCA-negative (N=73)		ANCA-positive (N=17)		p-value
Valve Involvement	Frequency	Percent	Frequency	Percent	
Missing Data	11	15.1	2	11.8	
Aortic Valve	19	26.0	6	35.3	0.549
Mitral Valve	30	41.1	5	29.4	0.422
Mitral and Aortic Valves	3	4.1	3	17.6	1.000
Mitral and aortic Valves with Implantable Cardioverter Defibrillator	1	1.4	0	0.0	1.000
Mitral, Aortic, and Tricuspid Valves	1	1.4	0	0.0	1.000
Mitral and Pulmonary Valve	1	1.4	0	0.0	1.000
Mitral and Tricuspid Valves	1	1.4	0	0.0	1.000
Tricuspid Valves	1	1.4	1	5.9	1.000
Implantable Cardioverter Defibrillator	5	6.8	0	0.0	0.579
Total Aortic	24	32.9	9	52.9	0.122
Total Mitral	37	50.7	8	47.1	1.000
Total Tricuspid	3	4.1	1	5.9	0.749
Implantable Cardioverter Defibrillator	6	8.2	0	0.0	0.866
Single Valve Involvement	50	68.5	12	70.6	

There were no statistically significant differences in laboratory characteristics, such as white blood cell count, CRP, or creatinine level, between patients with positive and negative ANCA antibodies (Table 4). Neither were there any significant differences in mortality or one-year mortality between patients with positive and negative ANCA antibodies (11.8% compared to 12.3%, P-value 1) (Table 5).

**Table 4:** Comparison of laboratory results in hospitalized patients with endocarditis ANCA: antineutrophil cytoplasmic antibodies; CRP: C-reactive protein; WBC: white blood cells

	ANCA-negative	ANCA-positive	p-value
	Median (IQR)	Median (IQR)	
Vegetation Size	4.00 (0.00 - 10.50)	0.00 (0.00 - 12.50)	0.639
CRP mean	69.00 (37.75 - 98.25)	48.50 (31.00 - 106.25)	0.465
CRP max	131.00 (77.00 - 218.00)	92.00 (57.00 - 188.00)	0.357
WBC max	13.00 (9.80 - 23.00)	12.40 (7.80 - 19.75)	0.375
WBC mean	8.75 (6.90 - 10.50)	6.70 (5.95 - 10.00)	0.116
Creatinine mean	1.19 (0.90 - 2.00)	1.40 (0.90 - 2.05)	0.451
Creatinine max	1.48 (1.00 - 3.30)	1.95 (1.25 - 3.68)	0.264

**Table 5:** Comparison of mortality, need of cardiovascular surgery, acute kidney injury, and length of hospitalization

6 months mortality	9 (12.3%)	2 (11.8%)	1.00
1-month Mortality	5 (6.8%)	1 (5.9%)	1.00
Cardiovascular Surgery	21 (28.8%)	6 (35.3%)	0.573
Acute Kidney Injury	34 (46.6%)	11 (68.8%)	0.167
Days of Hospitalization	15 (11-28)	18.5 (9.25-37)	0.740

Regarding secondary outcomes, patients with positive ANCA antibodies had a higher tendency toward acute kidney injury without statistical significance (68.8% compared to 46.6%, P-value 0.167). There were no statistically significant differences in the prevalence of heart surgery, one-month mortality, or days of hospitalization. Clinical characteristics of hospitalized subjects with endocarditis are shown in Table 6. 

**Table 6:** Clinical characteristics of hospitalized subjects with endocarditis ANCA: antineutrophil cytoplasmic antibodies

	ANCA-negative (N=73)		ANCA-positive (N=17)		p-value
	Frequency	Percent	Frequency	Percent	
Perforated Valve	8	11	2	12	1.000
Abscess	4	5	2	12	0.316
Duke Criteria	59	81	12	71	0.342
Fever	53	73	7	41	0.033
Rash	8	11	1	6	1.000
Vascular Phenomena	15	21	2	12	0.511
Immunologic Phenomena	6	8	0	0	0.590
Arthralgia/Arthritis	4	5	0	0	1.000
Urine protein	24	33	7	41	0.579
Urine Red Blood Cells	39	53	7	41	0.426
Urine Casts	23	32	5	29	1.000

In the multivariable analysis for six-month mortality, age was a statistically significant variable with an odds ratio of 1.069 and a P-value of 0.001, as well as the presence of a central line with an odds ratio of 17.7 and a P-value of 0.01.

## Discussion

In previous studies, the prevalence of ANCA-associated antibody positivity has been observed in patients suffering from infective endocarditis. The presence of ANCA positivity in patients with infective endocarditis poses a diagnostic and management challenge [17]. Many clinical symptoms associated with infective endocarditis, such as fever, weight loss, hepatosplenomegaly, and renal failure, overlap with those seen in patients with ANCA-associated vasculitis [18]. Consequently, in some cases, both diagnoses are concurrently under investigation, and determining which disease is the primary driver of organ injury becomes crucial for administering appropriate therapy.

Physicians faced with patients exhibiting ANCA positivity, infective endocarditis, and renal failure find themselves in the dilemma of deciding whether to supplement antibiotic treatment with immunosuppression. This decision is complex, requiring careful consideration of the underlying pathology and prioritizing the most effective therapeutic approach for the patient's overall well-being.

The findings of this study suggest that ANCA antibodies may be associated with specific microbiological agents, such as Q fever and *Enterococcus*, in patients with infectious endocarditis. This could have important implications for diagnosis and treatment strategies for these patients, as different microbiological agents may require different antibiotic regimens. Additionally, the higher prevalence of fever in ANCA-positive patients could also be a useful clinical indicator for infectious endocarditis.

However, the lack of statistically significant differences in six-month mortality between ANCA-positive and negative patients with infectious endocarditis may suggest that ANCA status alone may not be a strong predictor of clinical outcomes. Further research is needed to explore the complex relationship between ANCA antibodies, microbiological agents, and clinical outcomes in patients with infectious endocarditis.

As mentioned in the introduction, there are several hypotheses for the relationship between bacterial proteins and autoimmune diseases, including molecular mimicry and TLR activation. These mechanisms could potentially also be involved in the association between ANCA antibodies and infectious endocarditis, as bacterial proteins from infective endocarditis pathogens could trigger ANCA production through similar mechanisms [19].

Furthermore, the higher prevalence of vegetation in echocardiography in ANCA-positive patients with infectious endocarditis could be related to the role of ANCA antibodies in NET formation. NETs are known to contribute to the pathogenesis of infective endocarditis, and the binding of ANCA antibodies to neutrophils could potentially enhance NET formation, leading to increased vegetation formation [12].

This study had several limitations, including the relatively small sample size and retrospective design. Additionally, the study only included patients from a single center, which may limit the generalizability of the findings. Future studies with larger, multicenter cohorts and prospective designs could help validate these findings and provide more robust evidence for the association between ANCA antibodies and infectious endocarditis.

Furthermore, additional studies could explore the potential role of ANCA antibodies in the pathogenesis of infective endocarditis through in vitro and animal models. This could help to elucidate the mechanisms underlying the association between ANCA antibodies and infective endocarditis and could potentially lead to the development of new therapeutic targets for this condition.

## Conclusions

In conclusion, the necessity for additional research is paramount to ascertain whether ANCA positivity and its association with vasculitis in the context of infective endocarditis are indeed immunologic phenomena. Unraveling the specific mechanisms underlying this connection is of critical importance, not only for the development of targeted therapeutic interventions but also for identifying potential biomarkers that could enhance early detection and monitoring of the disease. Moreover, this research has the potential to contribute to a broader understanding of the intricate interplay between the immune system and infectious agents, offering insights that extend beyond endocarditis. The outcomes of such investigations hold promise for advancing clinical practices, improving patient outcomes, and shaping strategies for infectious disease management.
